# Successful Management of Aspirin Intolerance After Ad Hoc PCI: A Case Report and a Developed Algorithm

**DOI:** 10.1002/ccr3.71983

**Published:** 2026-02-06

**Authors:** Keyhan Mohammadi, Shakila Yaribash, Maryam Mehrpooya

**Affiliations:** ^1^ Department of Clinical Pharmacy, Faculty of Pharmacy Tehran University of Medical Sciences Tehran Iran; ^2^ Research Center for Antibiotic Stewardship and Antimicrobial Resistance, Imam Khomeini Hospital Complex, Tehran University of Medical Sciences Tehran Iran; ^3^ Faculty of Pharmacy Tehran University of Medical Sciences Tehran Iran; ^4^ Department of Cardiology School of Medicine, Imam Khomeini Hospital Complex, Tehran University of Medical Sciences Tehran Iran

**Keywords:** antiplatelet therapy, aspirin intolerance, case report, desensitization, ticagrelor

## Abstract

In managing atherosclerotic cardiovascular disease, especially after percutaneous coronary intervention (PCI), dual antiplatelet therapy (DAPT), prominently aspirin and a P2Y12 inhibitor, is fundamental. Nonetheless, aspirin hypersensitivity complicates treatment for some patients. Desensitization processes have been identified as a viable method to circumvent this issue. This case report describes a 75‐year‐old man diagnosed with significant stenosis in the coronary artery who was scheduled for elective PCI of the right coronary artery. The patient's medical record noted a hypersensitivity to aspirin. Initially, the patient underwent successful desensitization to aspirin, allowing for treatment with aspirin and Clopidogrel. However, the need for another desensitization emerged months later, which unfortunately was unsuccessful. As a result, the patient's treatment was shifted to Ticagrelor monotherapy, a potent antiplatelet strategy, which was carried out without any complications during the follow‐up period.

## Key Clinical Message

Aspirin hypersensitivity occurs in 1%–5% of patients and is often mistaken for intolerance, leading to undertreatment. Desensitization and low‐dose challenges are effective and safe but underutilized for patients needing aspirin despite hypersensitivity. If the desensitization fails, the selection of alternative antithrombotic therapy might be appropriate in selected cases.

## Graphical abstract

Proposed Algorithm for Management of Aspirin Intolerance After PCI.

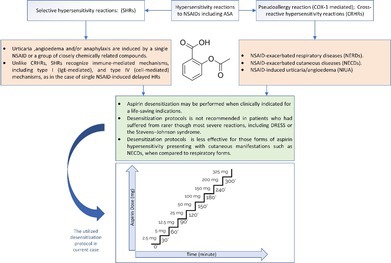

## 1 Introduction

Cardiovascular disease (CVD), including atherosclerotic cardiovascular disease (ASCVD), refers to the general manifestation of atherosclerosis and is the leading cause of global mortality and morbidity [1, 2]. Antithrombotic therapy, including antiplatelet therapy, is widely prescribed for the primary prevention and treatment of chronic coronary syndrome (CCS) and to decrease long‐term adverse ASCVD events [3]. Aspirin, a long‐standing mainstay of antithrombotic therapy in cardiovascular disease, remains essential in dual antiplatelet therapy (DAPT) regimens for patients undergoing percutaneous coronary intervention (PCI) [4, 5]. The standard duration of DAPT in CCS patients is six months, but it could be one to six months, depending on the risk of thrombosis and bleeding [6]. Whether assumed or proven, aspirin hypersensitivity presents a significant clinical challenge, restricting access to a critical life‐saving drug [7]. The prevalence of aspirin intolerance is estimated to affect 1% to 5% of the population [8]. Generally, the pathogenesis of aspirin intolerance and hypersensitivity reactions can be categorized into two pathways: IgE‐dependent mechanisms involving mast cells in the small bronchi of patients and COX enzyme inhibition, indigestion, and other pseudo‐allergic reactions [6]. These “pseudo‐allergic reactions”, which are usually known as aspirin intolerance, mainly due to COX‐1 inhibition and can be elicited by any nonsteroidal anti‐inflammatory drugs (NSAIDs) and can lead to various manifestations such as NISAD‐induced asthma and rhinosinusitis, NSAID‐induced urticaria/angioedema in patients with chronic urticaria, NSAID‐induced urticaria/angioedema in otherwise asymptomatic individuals and blended (mixed respiratory and/or cutaneous) reactions in otherwise asymptomatic individuals. These reactions can occur following exposure to all of the NSAIDs, especially with COX‐1 inhibitors, including ASA [9].

With its various successful protocols, aspirin desensitization offers a promising approach to overcoming hypersensitivity reactions and is a critical player in a life‐saving way [7, 10, 11]. Almost all protocols involve administering increasing doses of aspirin over short and fixed periods until the therapeutic dose is reached. If hypersensitivity reactions (HRs) occur during the procedure, symptomatic treatment is administered, and the patient is subsequently given the same dose of aspirin until they become tolerant [12]. After excluding other pseudo‐allergic reactions, such as gastrointestinal or indigestion due to aspirin, these protocols can be employed in most elective patients with a history of aspirin hypersensitivity reactions, especially those with a history of respiratory symptoms [7]. However, it is crucial to remember that desensitization is generally not recommended for severe allergic reactions, such as drug rash with eosinophilia and systemic symptoms (DRESS) or Stevens‐Johnson syndrome [7]. The literature reports the successful management of Aspirin hypersensitivity after PCI and, in addition, presents a new algorithm for this severe medical problem; however, there is not enough evidence to support the conclusion for these cases [13], especially in the setting of failure of desensitization protocol.

The case report describes a patient who underwent successful aspirin desensitization for elective PCI and was discharged with a maintenance dose of aspirin. However, the patient stopped taking aspirin about 3 months later. When the patient resumed aspirin a few days later, they experienced hypersensitivity reactions that required hospitalization. A second attempt at desensitization was unsuccessful due to factors such as the change in branding and the patient's different tolerance at a recent session. The patient was then successfully treated with ticagrelor monotherapy as a potent antiplatelet therapy and did not experience any reaction or ischemic event during the six‐month and one‐year follow‐up.

## 2 Case History and Examination

A 75‐year‐old man with a history of CCS has complained of an increase in the frequency of angina episodes during the recent few months. The patient was hospitalized for coronary angiography (CAG) and a possible PCI procedure on the coronary artery. The patient had a history of stable ischemic heart disease, diagnosed 20 years ago. He underwent CAG at that time and was subsequently treated with aspirin as antithrombotic therapy. However, he developed an intolerance to aspirin, experiencing respiratory and dermatologic reactions, including rash and pruritus. Patients experienced a recurrence of their hypersensitivity reactions to aspirin 2 years before the current event that was non‐severe. Therefore, based on past reactions to aspirin, the pharmacotherapy consultation was conducted to evaluate the patient's suitability for aspirin desensitization and to determine the appropriate desensitization protocol before CAG. The patient also reported experiencing dyspnea, potentially triggered by previous diclofenac ingestion. The patient did not report a history of asthma or nasal polyps, but he did experience episodes of allergic rhinitis. Based on this history, the patient was deemed suitable for aspirin desensitization. Laboratory data obtained on admission are presented in Table 1, which were within the normal range.

**TABLE 1:** Routine laboratory data on the admission.

Parameter	Result
White blood cell	4.7 × 10^9^ cells/L
Hemoglobin	14.3 g/dL
Platelet's count	161 × 10^9^ cells/L
Sodium	140 mEq/L
Potassium	3.7 mEq/L
Calcium	9 mEq/L
Phosphorus	2.8 mg/dL
Magnesium	1.9 mg/dL
Creatinine	1 mg/dL

## 3 Methods

**FIGURE 1:**
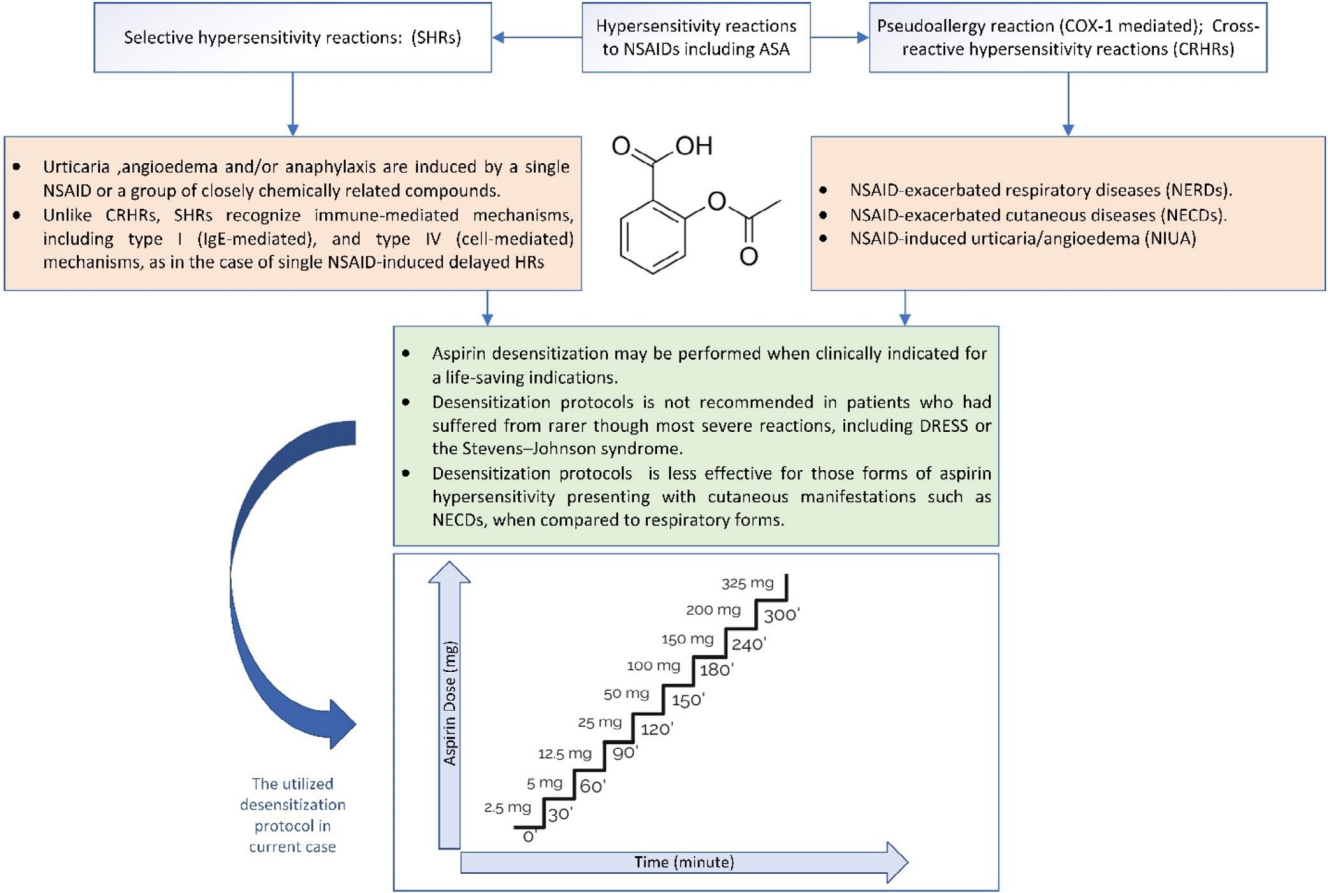
The infographic summary of aspirin desensitization and the protocol used in this case.

Following admission to the CCU, a desensitization protocol was implemented. This protocol, designed to induce tolerance to the aspirin, involved a gradual increase in dosage, as illustrated in Figure 1. No premedication was administered to the patient that could potentially interact with desensitization. Hemodynamic parameters, vital signs, and allergic reactions (including cough, bronchospasm, dyspnea, wheezing, and itching) were closely monitored throughout the desensitization procedure. The protocol will be followed if any symptoms occur, and appropriate symptomatic treatment will be administered. Once the symptoms resolve, the protocol can resume at the last administered dose. The final dose of aspirin (ASA) reached 325 mg; the typical dosage required before PCI. The desensitization protocol, conducted over 6 h, involved gradually increasing doses administered at 30–60‐min intervals. To administer lower doses of ASA (up to 12.5 mg), 100 mg non‐enteric coated ASA tablets were crushed and dispersed in 10 cc of distilled water, creating a 10 mg/cc concentration. This mixture was diluted further to achieve the desired lower 1 mg/cc concentration. Doses of 25 mg or more were administered by cutting 100 mg non‐enteric coated tablets (Table 2).

**TABLE 2:** Aspirin desensitization protocol.

Time (*T*, min)	ASA dose (mg)	Volume & dosage form	Route	Tolerated?
0	2.5	2.5 cc of 1 mg/mL	Oral	Yes
30	5	Five ccs of 1 mg/mL	Oral	Yes
60	12.5	12.5 cc of 1 mg/mL	Oral	Yes
90	25	One‐quarter of a 100 mg tablet	Oral	Yes
120	50	Half of a 100 mg tablet	Oral	Yes
150	100	One 100 mg tablet	Oral	Yes
180	150	One and a half 100 mg tablet	Oral	Yes
240	200	Two 100 mg tablet	Oral	Yes
300	325	One 325 mg tablet	Oral	Yes

*Note:* Nine sequential doses of aspirin (2.5, 5, 12.5, 25, 50, 100 m, 150, 200, and 325 mg) were administered orally for 6 h.

Abbreviation: ASA, acetylsalicylic acid.

The patient successfully tolerated the 325 mg aspirin dose on the first day. The following day, CAG and PCI were performed, and the patient underwent ad hoc PCI on LM‐LAD & RCA and was treated with aspirin and clopidogrel as antithrombotic therapy. The considerable stenosis in the LAD was deemed suitable for medical management. This included dual antiplatelet therapy (DAPT) with aspirin 80 mg daily and clopidogrel 75 mg daily for 6 months, a proton pump inhibitor (PPI), a high‐intensity statin, and a beta‐blocker. He was scheduled for a follow‐up in the following weeks. In addition to maintaining chronic desensitization, uninterrupted aspirin therapy is crucial after successful desensitization. If aspirin is discontinued for more than a few days, re‐initiation at regular doses may trigger intolerance and allergic reactions, necessitating another desensitization procedure [13]. At discharge, the patient was instructed to take aspirin 80 mg regularly once daily at a consistent time, without interruption, in combination with other drugs such as clopidogrel for 6 months, statin as well, and beta blocker at the maintenance therapy for anginal episodes. He was advised to contact his physician immediately if his aspirin dose was missed or interrupted for more than 2 days, as re‐desensitization may be required to re‐establish tolerance. The patient was advised to avoid taking unnecessary medications, including NSAIDs, without consulting with a healthcare provider.

## 4 Conclusion and Results

The patient returned to the hospital approximately 3 months after his PCI while still on DAPT. Despite instructions to maintain uninterrupted aspirin therapy for tolerance induction, he had missed 4 days of aspirin due to a local trip. Upon resuming aspirin on the second day, he developed symptoms, including nasal discharge, sneezing, nausea, vomiting, and a skin rash. He was readmitted for aspirin desensitization. The same hypersensitivity desensitization protocol was repeated, with the final dose adjusted to 80 mg for maintenance. However, during this process, at a dose of 50 mg, the patient developed severe nausea and vomiting, as well as sneezing, pruritus, and a mild rash on the trunk. At this stage, the patient was unwilling to take aspirin, stating that “the reactions to aspirin are bothersome to me, and I will never retake aspirin.” He declined further attempts at desensitization to continue taking aspirin. Therefore, aspirin was discontinued for the patient. Considering his previous stenting, involvement of other coronary arteries (including stenosis of the left main coronary artery), and his low bleeding risk, monotherapy with a more potent P2Y12 inhibitor, such as ticagrelor, was recommended instead of clopidogrel monotherapy [6]. Ticagrelor was administered at 90 mg twice daily, 24 h after the last dose of clopidogrel. The patient tolerated the medication without any side effects, and his allergic symptoms subsided the following day. At the three‐month follow‐up, the patient was taking ticagrelor without any bleeding or ischemic complications.

## 5 Discussion

As an antithrombotic therapy, aspirin is typically prescribed long‐term for patients with CCS; however, it is crucial to consider individual patient factors, such as bleeding risk and hypersensitivity, when prescribing aspirin for CCS management [6, 14]. The latest 2024 ESC guidelines for CCS recommend low‐dose aspirin (75–100 mg once daily) as the standard treatment for CCS patients, regardless of whether they have had a previous myocardial infarction (MI). Clopidogrel monotherapy is a safe and effective alternative to aspirin monotherapy for long‐term secondary prevention in these patients. In select patients at high ischemic risk but without high bleeding risk (HBR), ticagrelor monotherapy may be considered based on the last guideline. For CCS patients undergoing PCI, a standard DAPT duration of 6 months is recommended; however, shorter DAPT durations (1 or 3 months) can be recommended to decrease the risk of bleeding [6].

The European Academy of Allergy and Clinical Immunology (EAACI) [15] classifies hypersensitivity reactions (HRs) to nonsteroidal anti‐inflammatory drugs (NSAIDs) into two categories: Cross‐reactive HRs (CRHRs): Triggered by chemically unrelated NSAIDs, these reactions are related to the inhibition of COX enzymes and selective HRs (SHRs): Induced by one or more NSAIDs within the same chemical group, these reactions involve immune‐mediated mechanisms (IgE or T cell‐mediated). Three main clinical phenotypes of cross‐reactive hypersensitivity reactions include NSAID‐exacerbated respiratory diseases (NERDs), NSAID‐exacerbated cutaneous diseases (NECDs), and NSAID‐induced urticaria/angioedema (NIUA). Inhibition of COX‐1 and COX‐2 enzymes by NSAIDs reduces prostaglandin (PG) synthesis, particularly PGE2, leading to a shift in arachidonic acid (AA) metabolism towards the production of cysteinyl leukotrienes (cysts), such as LTC4, LTD4, and LTE4. This shift contributed to bronchial hyperreactivity and exacerbated nasal symptoms [7]. Aspirin‐exacerbated respiratory disease (AERD), which includes asthma symptoms and rhinitis/nasal polyps, is a respiratory reaction such as NERDs to aspirin [16, 17]. On the other hand, aspirin and other NSAIDs can induce selective hypersensitivity reactions (SHRs) that lead to immediate or delayed onset of symptoms without cross‐reactivity with other NSAIDs. These reactions exhibit variable clinical presentations attributed to immunological mechanisms (not COX inhibition) involving IgE‐mediated or cell‐mediated pathways [18]. Additionally, it should be remembered that patients can experience a pharmacological reaction on one occasion and an immunological response on another [10].

The primary mechanism is the inhibition of the COX‐1 enzyme, which can lead to increased leukotrienes and decreased prostaglandins. Because of this, there is a cross‐reaction between aspirin sensitivity and NSAIDs [19]. Non‐allergic Aspirin sensitivity includes two main types: the bronchospastic and the urticarial/angioedema presentations [15]. Immune‐mediated Aspirin allergy could be the direct reaction of the immune system or to the molecule. The acute form, called the single‐NSAID‐induced urticaria/angioedema or anaphylaxis (SNIUAA), is an IgE‐mediated allergic response. A delayed reaction is called the single‐NSAID‐induced delayed hypersensitivity reaction (SNIDHR) that occurs more than 24 h after exposure. The range of clinical presentations varied from a skin rash to severe complications, including Stevens‐Johnson/toxic epidermal necrolysis [20]. Three significant scenarios occur clinically in hypersensitivity reactions: AERD (the triad of rhinitis, asthma, and nasal polyps), cutaneous manifestations (including urticaria and angioedema), and systemic reactions. Aspirin hypersensitivity in patients with asthma disease is reported to be 5%–24% of cases [7]. Pseudo‐allergy’ with Aspirin is also often reported with different symptoms (such as aseptic meningitis) [7].

Many desensitization protocols have been developed over the years. A slow protocol, known as the Scripps protocol, is a 3‐day procedure involving gradual dose escalation of aspirin every 3 h, starting from 30 mg and reaching up to 650 mg. In contrast, the rapid Wong protocol involves dose escalation over a shorter timeframe of 85 to 135 min [21, 22].

A clinical study investigated responses to chronic oral Aspirin desensitization in 20 cases with aspirin‐induced and 14 patients with aspirin‐tolerant asthma [23]. All participants reported chronic rhinosinusitis and nasal polyposis. Patients were randomly assigned to receive 624 mg of aspirin or placebo once daily for 6 months. The results showed that only one patient with aspirin‐induced asthma experienced clinical improvements in nasal and bronchial symptoms.

In a multicenter observational study, 330 patients with a history of Aspirin sensitivity underwent coronary angiography with intent to PCI. Their adverse effects to Aspirin varied from dermatology presentation to anaphylactic reaction. They underwent a rapid (5.5 h) Aspirin desensitization protocol before cardiac catheterization except in patients with ST‐segment–elevation myocardial infarction in whom the desensitization was performed after primary PCI. In 95.4% of cases, the desensitization procedure and all patients who experienced anaphylactic reactions before were successful. Adverse effects in patients who did not successfully respond to the desensitization were minor and managed by corticosteroids and antihistamines. Most patients with successful desensitization continued aspirin for at least 12 months [24].

A case report by Ahmed‐Khan et al. describes the complex situation of a 50‐year‐old male who presented to the emergency department with a diagnosis of ST‐elevation myocardial infarction [25]. The patient also had a history of asthma and AERD, a condition that can trigger severe asthma attacks with even minimal aspirin exposure. Following the administration of high doses of aspirin, a standard treatment for MI, the patient developed an asthma exacerbation. To ensure he could receive the necessary aspirin therapy for his MI, the medical team implemented the Wong protocol, also known as the rapid protocol. This involved gradually increasing the aspirin dosage over a controlled period, allowing the patient's body to become accustomed to the medication. This case report highlights the significant challenges faced by patients with AERD who require aspirin for other medical conditions.

In another case report involving a hypersensitivity reaction to antithrombotic therapy, the authors successfully switched the patient's medication from rivaroxaban to apixaban due to a cutaneous rivaroxaban‐induced response [26].

Risk factors of failure in aspirin desensitization in a retrospective study were evaluated. An index reaction of angioedema to NSAIDs and atopic disease as comorbidity was significantly associated with a higher risk of failing in the aspirin desensitization process [27]. As mentioned earlier, while numerous reports document successful aspirin desensitization across various types of aspirin hypersensitivity reactions, including AERD, a significant gap exists in the data regarding patients who require re‐desensitization.

This report presents a patient who was a candidate for CAG and elective percutaneous coronary intervention with a history of coronary artery disease (CAD) and previous hypersensitivity reactions to aspirin and diclofenac. These reactions included itching, rash, shortness of breath, and sneezing upon exposure to these medications. Consequently, a desensitization protocol was implemented before angiography and PCI, involving gradual increases in aspirin dosage to reach a loading dose of 325 mg. The initial desensitization protocol proved successful, achieving a temporary tolerance to aspirin. The patient was advised to take a daily maintenance dose of 80 mg of aspirin at discharge.

The patient temporarily discontinued aspirin for a few days due to a local trip after 3 months of successful maintenance therapy. Upon resuming aspirin, they experienced a recurrence of their hypersensitivity reactions, including mild rash, itching, and gastrointestinal symptoms, despite prior successful desensitization. The patient was a candidate for re‐desensitization. In this session, a desensitization protocol, mirroring the previous one, was implemented to achieve a maintenance dose of 80 mg aspirin per day. However, this attempt was unsuccessful, resulting in significant adverse effects. The patient experienced severe gastrointestinal reactions indicative of a pronounced sensitivity to aspirin—additionally, mild dermatological symptoms presented, characterized by a rash and itching (pruritus). Furthermore, rhino‐orbital manifestations were observed, consisting of sneezing, excessive tearing (lacrimation), and a rash affecting the nose and eye area. The recurrence of the allergic reaction, despite initial successful treatment, strongly suggests the presence of a more complex and persistent underlying mechanism driving the patient's sensitivity to aspirin. This persistent sensitivity is not simply a surface‐level phenomenon but points to a more profound, potentially intrinsic, biological factor contributing to the allergic response. This recurring intolerance has significant implications for patient care. Not only does it create a persistent risk of adverse reactions, but it also leads to the patient's reluctance to engage with a potentially life‐saving medication. Even when medically indicated, the patient's hesitation to take aspirin underscores the profound impact these allergic reactions have on their quality of life and ability to manage their health effectively. Given the underlying mechanism's complexity and the allergic response's significant consequences, a comprehensive exploration of alternative treatment strategies is crucial. This exploration should encompass a multi‐faceted approach, considering the immediate relief of symptoms and the underlying biological factors contributing to the patient's sensitivity. Such an approach will enable the development of more effective and long‐term solutions, mitigating the risks of future allergic reactions and ensuring the patient's safe and successful management of their health conditions. Therefore, aspirin was discontinued, and due to pharmacotherapy consultation, due to the high ischemic risk of the patient [6], clopidogrel was switched to a more potent antiplatelet, ticagrelor 90 mg twice daily, beginning 24 h after the last dose of clopidogrel.

## 6 Conclusion

The management of atherosclerotic coronary artery disease requires DAPT after PCI. However, aspirin hypersensitivity poses challenges, particularly early post‐PCI. This case study presents a 75‐year‐old experiencing stenosis and allergic reactions to aspirin. After initial desensitization success, a second procedure failed, necessitating Ticagrelor monotherapy instead. Consequently, individualized therapies offer promising alternatives for those struggling with aspirin intolerance or resistance. Thus, tailored approaches may significantly enhance care quality and clinical outcomes.

## Author Contributions

**Keyhan Mohammadi:** conceptualization, data curation, formal analysis, funding acquisition, investigation, methodology, project administration, resources, supervision, validation, visualization, writing – original draft, writing – review and editing. **Shakila Yaribash:** conceptualization, investigation, methodology, project administration, software, supervision, visualization, writing – original draft, writing – review and editing. **Maryam Mehrpooya:** conceptualization, data curation, formal analysis, funding acquisition, methodology, software, supervision, validation, writing – review and editing.

## Funding

The authors have nothing to report.

## Consent

Written informed consent was obtained from the patient to publish this report in accordance with the journal's patient consent policy.

## Conflicts of Interest

The authors declare no conflicts of interest.

## Data Availability

All data generated or analyzed during this study are available as part of the article, and no additional source data are required.
